# Bispecific Antibodies for the Management of Relapsed/Refractory Multiple Myeloma

**DOI:** 10.3390/cancers16132337

**Published:** 2024-06-26

**Authors:** Paola Tacchetti, Simona Barbato, Katia Mancuso, Elena Zamagni, Michele Cavo

**Affiliations:** 1 IRCCS Azienda Ospedaliero-Universitaria di Bologna, Istituto di Ematologia “Seràgnoli”, Bologna, Italy; paola.tacchetti2@unibo.it (P.T.); simona.barbato3@unibo.it (S.B.); katia.mancuso3@unibo.it (K.M.); e.zamagni@unibo.it (E.Z.); 2 Dipartimento di Scienze Mediche e Chirurgiche, Università di Bologna, Bologna, Italy

**Keywords:** multiple myeloma, immunotherapy, bispecific antibodies

## Abstract

**Simple Summary:**

Bispecific antibodies (BsAbs) provide a new mode of targeting multiple myeloma (MM) plasma cells (PCs), basing their action on the activation and redirection of the T-cell compartment against neoplastic cells. Two BCMA-targeting (teclistamab and elranatamab) plus one GPRC5D-targeting (talquetamab) BsAbs are available for the management of heavily pretreated patients with relapsed/refractory (RR) MM. Novel strategies to augment potency, reduce toxicity, and improve management are under investigation. This review summarizes the clinical applications of BsAbs and discusses the current challenges of the treatment and opportunities for optimization.

**Abstract:**

Bispecific antibodies (BsAbs) are artificially engineered antibodies that can bind simultaneously to the CD3 subunit within the T-cell receptor complex and an antigen on tumor cells, leading to T-cell activation and tumor cell killing. BsAbs against BCMA or GPRC5D have shown impressive clinical activity in heavily pretreated patients with relapsed/refractory multiple myeloma (RRMM), with some agents having already received regulatory approval after the third (by the European Medicines Agency, EMA) or fourth (by the Food and Drug Administration, FDA) line of therapy; the results of early-phase clinical trials targeting FcRH5 are also promising. Overall, BsAbs as monotherapy correlated with an ORR that exceeded 60%, with a high CR rate ranging between 25% and 50% and a median PFS of around 1 year among patients with a median of 4–6 prior lines of therapy. The main toxicities include cytokine release syndrome, cytopenias, hypogammaglobulinemia, and infections; on-target off-tumor adverse events involving the skin, mucosa, hair, and nails may also occur with anti-GPRC5D BsAbs. Active research to increase their efficacy and improve their tolerance is still in progress, including combination therapies and application in earlier treatment lines and the development of novel agents. A better understanding of the mechanisms of resistance is a challenge and could lead to more personalized approaches.

## 1. Introduction

Multiple myeloma (MM) is the second most prevalent hematological malignancy, accounting for 10% of hematologic malignancies and 0.9% of all cancers. It is characterized by the clonal proliferation of plasma cells (PCs), production of monoclonal protein biomarkers, and possible organ dysfunction [1].

The pathogenesis of MM is complex and is characterized by different genetic alterations as driving events, genomic instability, and clonal evolution [2]. The complex interaction between mesenchymal stromal cells (MSCs) and MM cells leads to an overproduction of cytokines and growth factors that support and sustain tumor growth, progression, and therapy resistance. In this context, immune dysfunction, due to an altered T-cell repertoire with features of terminally differentiated T cells and loss of antigen-specific T-cell function, as well as ineffective antigen processing/presentation by tumor cells or dendritic cells, plays a crucial role in MM PCs survival [3,4].

Improved knowledge of the disease biology has led to the development of new strategies and treatment approaches. The introduction of proteasome inhibitors (PIs), immunomodulatory drugs (IMiDs), and monoclonal antibodies (mAbs) in recent decades has resulted in enhanced rates and depth of durable responses, reaching a median overall survival (OS) exceeding 8.5 years [5]. However, despite these treatment advances, treatment-resistant clones may emerge, and most patients experience cycles of remission and relapse. Particularly, relapsed/refractory MM (RRMM) patients already exposed to PIs, IMiDs, and anti-CD38 mAbs (referred to as triple-class exposed, TCE) have a poor prognosis using standard treatments, with a median progression-free survival (PFS) of approximately 4.5 months and a median OS slightly higher than 1 year [6], supporting a need for treatments with novel mechanisms of action.

New treatments aimed at redirecting T lymphocytes to act directly against tumor cells and overcoming the immunosuppressive microenvironment are changing this scenario. To date, three bispecific antibodies (BsAbs) and two chimeric antigen receptor (CAR) T-cell products (i.e., ide-cel and cilta-cel) are available for the management of TCE RRMM and represent new standards of care in heavily pretreated diseases [7,8,9,10,11]. Moreover, active research in this field is still in progress to develop newer constructs and evaluate their use in earlier lines of therapy and/or in combination strategies. These new therapeutic approaches are leading to a treatment paradigm shift and will greatly further improve the outcomes of MM patients. 

In this review, we will focus on the mechanisms of action, major targets, main results achieved so far, and future directions of BsAbs therapies in MM.

## 2. BsAbs Mechanisms of Action

BsAbs are engineered artificial antibodies that are able to activate T cells upon binding to target-expressing cells. Basically, CD3-BsAbs are designed to concomitantly bind the CD3 on T cells and a tumor antigen expressed on the surface of MM PCs; the membrane proximity caused by this close cell-cell apposition creates an immunological synapse between the immune effector cell and the malignant cell, which leads to the release of molecules that stimulate T-cell receptor (TcR) signaling and, downstream, induce T-cell activation (mainly CD8+ T cells). TcR signaling includes various signaling cascades, such as the nuclear factor kappa B (NF-κB) pathway, the Ras–extracellular signal-related kinase (ERK) pathway, and the inositol triphosphate/protein kinase B/mammalian target of rapamycin (IP3/Akt/mTOR) pathway, among others. Though the molecular mechanism that induces T-cell activation upon linkage with BsAbs has not been fully described, it has been shown that the efficiency of synapse formation correlates with the proximity of the binding epitope to the cell membrane [12]. T-cell activation is then accompanied by the secretion of granzymes and perforins release, cytolytic molecules that cause MM cell lysis and further induce the activation, proliferation, and differentiation of T cells, along with the production of different cytokines such as interleukin (IL)-6, IL-2, interferon-gamma (INF-γ), IL-5, and monocyte chemoattractant protein-1 (MCP-1), eventually leading to MM PCs killing. Notably, this immunomodulatory effect is not dependent on antigen presenting cells or major histocompatibility complex (MHC), and this aspect is noteworthy in the context of a dysfunctional immune system related to MM [13,14,15]. 

BsAbs were initially developed as recombinant proteins composed of two different single-chain variable fragments without the functional constant region fragment (Fc) and connected by a linker, named bispecific T-cell engagers (BiTEs). Due to their small size, BiTEs easily penetrate tumors but are characterized by a short half-life, plaguing patients with frequent or continuous infusions [16]. Conversely, modern engineered BsAbs have an IgG-like structure with two different single-chain variable fragments linked by an Fc region (DuoBody). The Fc domain of IgG-like BsAbs prolongs the elimination half-life by increasing the size of the molecule and by enabling binding to the neonatal Fc receptor (FcRn), which recycles IgG, allowing for intermittent rather than continuous dosing. The Fc portion also confers further properties, including antibody-dependent cellular cytotoxicity (ADCC) and complement-dependent cytotoxicity (CDC), although these effector functions can be silenced through modifications during drug development to prevent specific T-cell activation and excessive cytokines secretion, leaving an extended half-life as the major distinction. Silencing Fc regions through the removal of Fc gamma receptor (FcɣR) binding by site-specific mutations is an approach to retain the FcRn-driven half-life extension while reducing adverse immune induction [17,18]. All recent studies on BsAbs include Fc-containing products.

## 3. BsAbs Targets

BsAbs act via a double link with CD3 on T cells and a tumor-associated antigen expressed at the PC level. Ideal targets on the PC should have selective expression on malignant cells, with minimal or no expression in healthy tissues, to maximize tumor cell killing while having minimal side effects deriving from non-target toxicities. In this sense, tumor-specific antigens (TSAs) that are produced and differentially expressed by tumor cells and not by normal tissues would represent the best candidates. However, most antigens expressed on malignant cells may also be found on normal cells, and this may be the basis for potential “on-target/off-tumor” toxicities. 

Moreover, these targets should play a key role in proliferation-, survival-, and maturation-related pathways. So far, major targets in MM include the B-cell maturation antigen (BCMA), G protein-coupled receptor class C group 5 member D (GPRC5D), and Fc receptor-like 5 (FcRH5) (Table 1 for more details). 

BCMA, also known as tumor necrosis factor receptor superfamily 17 (TNFRSF17) or CD269, is a type III transmembrane glycoprotein that is expressed on mature B-lymphocytes, overexpressed on malignant PCs, and absent in non-hematological tissues. BCMA binds to B-cell-activating factor (BAFF) and a proliferation-inducing ligand (APRIL) and activates antiapoptotic pathways, including NF-kB, mitogen-activated protein kinase (MAPK8/JNK), and protein kinase B (Akt/PKB), with the transcription of anti-apoptotic Bcl-2 family members and other genes involved in cell survival and implicated in pro-tumorigenic function. In this way, BCMA has a fundamental role in the maturation and differentiation of B-lymphocytes, and its overexpression on MM PCs promotes tumor survival, growth, resistance to apoptosis, adhesion, and angiogenesis [19,20,21,22,23]. Moreover, APRIL directly impacts T-regulatory (Treg) cells, an aspect that may have a negative impact on the BsAbs-mediated killing of MM cells. The level of BCMA expression on neoplastic PCs can vary among patients and even within a patient based on the disease course and therapeutic pressure [14]. Currently, BCMA is the most explored immune target for BsAbs but also for CAR T cells and antibody–drug conjugates (ADC) in the treatment of MM.

GPRC5D is an orphan G-protein coupled receptor with an unknown ligand and not-yet-established signaling mechanism and function. GPRC5D protein has been predominantly detected in immune cells with a PC phenotype and in cells producing hard keratin, such as cortical cells of the hair shaft, the keratogenous zone of the nail, and filiform papillae of the tongue [24,25,26]. Like BCMA, GPRC5D is upregulated in neoplastic PCs, and increased GPRC5D mRNA expression from bone marrow or MM cells is associated with a high number of genetic aberrations and high-risk disease [25,27].

FcRH5—also known as FcRL5, IFGP5, BXMAS1, CD307, or IRTA2—is a membrane surface protein related to the group of receptors homologous to FcɣR 1 (FcɣRI), whose function is not yet fully understood. FcRH5 is expressed only in B cells, with an increasing expression in mature B cells and PCs. Moreover, FcRH5 expression is significantly higher in MM PCs than in normal PCs, and the expression levels of the FcRH5 protein have been reported to be significantly elevated in the patients with 1q21 alterations, being the FcRH5 gene located on the human chromosome 1 band 1q23.1 [28,29,30].

## 4. BCMA-Targeting BsAbs

AMG420, a BCMAxCD3 BiTE, was the first-in-class BsAb that demonstrated a significant activity in the treatment of heavily pretreated RRMM; however, due its short half-life requiring continuous intravenous (IV) infusion, this compound was not further developed [31]. Currently, two IgG-like BCMAxCD3 BsAbs, teclistamab [7,32] and elranatamab [8], received regulatory approval for the treatment of TCE RRMM. Moreover, several other BCMA-targeting BsAbs are in clinical development (Table 2).

### 4.1. AMG-420

AMG-420 is a BCMAxCD3 BiTE. In the phase 1 first-in-human dose-escalation trial (NCT02514239), a total of 42 RRMM patients, who had progression after at least two lines of therapy including PIs and IMiDs, received up to 10 cycles of AMG 420 at a dose of 0.2 to 800 mg/die as a continuous IV infusion every 4 weeks of a 6-week cycle. The overall response rate (ORR) was 31% in the entire cohort, and it was 70% at the maximum tolerated dose of 400 mg/die, including 50% (5 out of 10 patients) minimal residual disease (MRD)-negative complete responses (CR). Grade ≥ 3 adverse events (AEs) included infection (19%), peripheral polyneuropathy (5%), edema (2%), and cytokine release syndrome (CRS) (2%) [31]. These data provided an important proof-of-concept for BCMA-directed BsAbs in the treatment of RRMM; however, the logistical challenges of the continuous IV infusion led to the discontinuation of the development of this product in favor of half-life-extended BCMA-targeting BsAbs.

### 4.2. Teclistamab

Teclistamab (JNJ-64007957) is a humanized BCMAxCD3 IgG-like BsAb that contains mutations in the Fc region to stabilize and minimize its immunological effector functions. It was the first approved BsAb in MM based on the first-in-human phase 1/2 trial MajesTEC-1 (NCT04557098; NCT03145181) [7,32]. On August 2022, teclistamab received conditional marketing authorization by the European Medicines Agency (EMA) for the treatment of adult TCE RRMM patients who have received at least three prior therapies and have demonstrated disease progression on the last therapy, while on October 2022, the Food and Drug Administration (FDA) granted accelerated approval for the treatment of TCE RRMM for those who have previously received at least four prior therapies.

The first-in-human dose-escalation phase 1 MajesTEC-1 trial (NCT03145181) enrolled 157 patients with RRMM who were relapsed, refractory, or intolerant to established therapies. Teclistamab was administered IV (range 0.3−19.2 µg/kg [once every two weeks] or 19.2−720 µg/kg [once per week]) or subcutaneous (SC) (range 80−3000 µg/kg [once per week]) in different cohorts, with step-up dosing for 38.4 µg/kg or higher doses, and the recommended phase 2 dose (RP2D) was established as a weekly SC injection of 1.5 mg/kg with two step-up doses (0.06 mg/kg and 0.3 mg/kg) [32]. In the phase 1/2 portions of the study (NCT03145181; NCT04557098) a total of 165 patients received the RP2D [7]. The median prior lines of therapy was 5 (range 2 to 14), and all patients were TCE, 78% of whom had triple-class refractory (TCR) disease, while 26% had high-risk cytogenetics, and 17% had extramedullary disease (EMD). At a median follow-up of 14.1 months, the ORR was 63%, including 39% of patients who achieved at least a CR, and the median duration of response (DoR) and PFS were 18.4 and 11.3 months, respectively [7]. The updated analysis of the study with a median follow-up of 23 months reported an ORR of 63%, with 45.5% ≥CR and MRD negativity (10–5 sensitivity) by day 100 in 34 out of 42 (81%) evaluable patients. The median (m) PFS was 11.3 months (26.7 months for patients achieving ≥ CR), while the mDoR and mOS were 21.6 months and 21.9 months, respectively [33]. Common AEs included neutropenia (71% of patients, 65.5% grade ≥ 3), anemia (54.5%, with 38% grade ≥ 3), and thrombocytopenia (41%, with 22% grade ≥ 3). CRS was observed in 72% of the patients, almost always grades 1–2 (less than 1% grade 3 CRS, no grade 4), while the rate of immune effector cell-associated neurotoxicity syndrome (ICANS) was low (3%, all grade 1 or 2). Also, hypogammaglobulinemia and lymphopenia were found (in 21% and 36% of patients, respectively), ultimately leading to frequent infections, which were reported in 80% of the patients (including 55% being grade 3 or 4 and 13% being grade 5). Among infections, those of grade ≥ 3 were mainly related to COVID-19 (33%), respiratory infections (21%), and Pneumocystis jirovecii pneumonia (PJP, 4%) [7,33]. Notably, in the MajesTEC-1 trial patients who had achieved a confirmed partial response (PR) or better after four or more cycles of treatment (phase 1) or a confirmed CR or better for six months or longer (phase 2) were eligible to reduce the dosing frequency to 1.5 mg/kg SC every two weeks. Of the 104 out of 165 responders who had received teclistamab at the RP2D, 63 patients switched to bi-weekly dosing. The median time to switch was 11.3 months (range 3–30), the majority (86%) of patients who switched to a bi-weekly scheduling were in CR or better, and 69% remained in response for at least two years from the time of their first response at a median follow-up of 12.6 months after switching. Interestingly, after 12–18 months of follow-up, the new onset of grade ≥ 3 infections was lower in responders who switched to bi-weekly dosing on or before 12 months than those who remained on weekly dosing at 12 months (16% vs. 33%) [34]. Based on these results, in August 2023, the European Commission (EC) granted the approval of a variation application for teclistamab, providing the option for a reduced dosing frequency of 1.5 mg/kg every two weeks for patients who have achieved at least a CR for a minimum of six months.

The efficacy and safety of teclistamab was also explored in patients previously exposed to anti-BCMA treatments. Specifically, 40 patients enrolled in cohort C of the MajesTEC-1 trial were previously treated with ADC (72.5%) or CAR-T (37.5%), and the resultant ORR was similar (52.5%) in the two subgroups, albeit slightly lower than in anti-BCMA-naïve patients, while the toxicity profile did not show any new concern for safety, supporting the possibility of using teclistamab as an effective therapy for patients with prior exposure to BCMA-targeted agents [35].

The real-world safety and efficacy of teclistamab have been evaluated in different retrospective analyses including RRMM patients who received teclistamab as the standard of care [36,37,38]. Despite more heavily pretreated patient populations compared to the MajesTEC-1 trial—with six median prior lines of therapy for more than 85% of the patients with TCR disease and between one-third and one-half of patients who received prior BCMA targeted therapy—the ORR was similar to that which was reported in the MajesTEC-1 trial, with approximately two-thirds of patients (59–66%) achieving at least a PR, while the rate of CR or better was slightly reduced (20–29%), and the mPFS was between 5.6 and 8.7 months. The incidence of severe CRS (1–3.5%) and ICANS (1–4.5%) was low. Also, interesting preliminary data on the prophylactic use of Tocilizumab prior to the administration of the first, or second, step-up dose of teclistamab seem to be associated with a reduced incidence of CRS, retaining responses comparable to those observed in the MajesTEC-1 study [39,40]. The risk of cytopenias (more than half of patients experienced grade ≥ 3 events) and related infections (observed in one-third or one-half of patients, in approximately one-quarter grade ≥ 3) remained an ongoing challenge; however, primary prophylaxis with intravenous immunoglobulin (IVIG) was associated with a significantly lower infection risk. Overall, these real-world data showed that teclistamab was safe and effective in a real-life setting, even though most patients did not meet the eligibility criteria for the MajesTEC-1 trial.

### 4.3. Elranatamab

Elranatamab (PF-06863135) is a humanized BCMAxCD3 IgG-like, Fc-modified BsAb. In August 2023, the FDA granted accelerated approval to elranatamab for the treatment of TCE RRMM in those who have previously received at least four lines of therapy, and in December 2023, the EMA gave a positive recommendation for conditional marketing authorization for the treatment of TCE RRMM in those who have previously received at least three prior therapies [8].

Data from the first-in-human phase 1 trial MagnetisMM-1 (NCT03269136) on 58 RRMM patients receiving SC elranatamab as single agent at doses starting from 215 up to 1000 µg/kg either weekly or every two weeks demonstrated encouraging safety and efficacy results, with an ORR of 64%, including 38% of at least CR, and mDoR and mPFS of 17.1 and 11.8 months, respectively [41]. In the registrational phase 2 MagnetisMM-3 study (NCT04649359), TCR MM patients received two step-up priming doses of elranatamab (of 12 mg and 32 mg) and then the RP2D of 76 mg once weekly SC in 28-day cycles for six cycles. Thereafter, persistent responders switched to a one-dose-every-two-weeks regimen. A total of 123 patients without prior BCMA-directed therapy (with five median prior lines of therapy [range 2–22], 97% TCR disease, 25% high-risk cytogenetics, and 32% EMD) were enrolled in cohort A of the study. The ORR was 61% (35% ≥ CR) upon 14.7 months of median follow-up, and MRD negativity (10−5 sensitivity) in ≥CR patients was reported in 90% (*n* = 29) of evaluable patients. Notably, the responses were confirmed, although a lower ORR was found in patients with EMD (38.5%) or revised international staging system (R-ISS) stage III (26%). The DoR, PFS, and OS rates at 15 months were 71.5%, 51% (90% in patients who achieved CR), and 57%, respectively. With an extended follow-up, the mPFS and mOS were 17.2 and 21.9 months, respectively [42]. Common AEs included hematological AEs, infections, and CRS, comparable to those reported with teclistamab. Anemia was observed in 49% of the patients (37% grades 3–4), neutropenia in 49% (all grades 3–4), and thrombocytopenia in 31% (24% grades 3–4). Infections were observed in 70% of patients, including 40% grades 3–4 and 6.5% fatal infections, and the most frequently reported were COVID-19 related (29%). Immune paresis was observed in 99% of the patients at baseline, and an IgG level < 400 mg/dL at least once during the treatment period was reported in 75.5% of the patients. CRS occurred in 58% of the patients, all grade 1 or grade 2, while ICANS occurred in only 3% of the patients, all grade 1 or 2. Notably, among responders who switched to bi-weekly dosing, 80% of patients maintained or improved their response for at least 6 months, while the incidence of grade 3 or 4 AEs decreased from 59% to 47%, suggesting that a reduced dose intensity may improve the long-term safety without compromising efficacy [8].

In a pooled analysis of four MagnetisMM trials (NCT03269136, NCT04798586, NCT04649359, and NCT05014412), the efficacy and safety of elranatamab monotherapy were evaluated in RRMM patients previously exposed to anti-BCMA therapies. Data from 87 patients showed an ORR of 46%, including 18% of at least CR, and mPFS of 5.5 months. Specifically, patients who received a prior BCMA-directed ADC (*n* = 59) showed an ORR of 42% and mPFS of 3.9 months, while those who had prior treatment with a BCMA-directed CAR-T therapy (*n* = 36) achieved an ORR of 53% and mPFS of 10.0 months. Overall, these data are encouraging to promote further research on the use of elranatamab after prior anti-BCMA therapies [43].

### 4.4. Linvoseltamab

Linvoseltamab (REGN5458) is a fully human BCMAxCD3 BsAb. This drug also possesses an anti-albumin domain in the Fc region, which allows for a reduced dosing frequency. The phase 1/2 LINKER-MM1 trial (NCT03761108) explored the use of IV linvoseltamab at the dose of 50 (*n* = 104 patients) or 200 mg (*n* = 117 patients) in RRMM patients who were TCR or TCE and progressed after at least three lines of therapy. The median prior lines of therapy was 5 (range 1–16), and 81% of the patients were TCR. At the recommended dose of 200 mg (with two step-up doses of 5 and 25 mg), linvoseltamab showed a deep response, with an ORR of 71%, including 30% of at least CR. A high ORR (≥50%) was observed across high-risk subgroups, including patients aged ≥ 75 years old, with ISS stage II–III, with EMD, with elevated levels of soluble BCMA at baseline, or high cytogenetic risk. Across the 50 and 200 mg cohorts, 54.3% of patients with CR or better and evaluable samples (*n* = 46) were MRD negative at 10^−5^. At the recommended dose of 200 mg, the probability of PFS at 12 months was 66%. Linvoseltamab showed a generally manageable safety and tolerability profile, and the most relevant AEs at 200 mg were CRS, 45% (only 1% grades 3–4); neutropenia, 32.5% (31% grades 3–4); anemia, 27% (24% grades 3–4); thrombocytopenia, 17% (14% grades 3–4); infections, 60% (37% grade ≥ 3); and ICANS 6% (3% grades 3–4) [44,45].

### 4.5. Alnuctamab

Alnuctamab (CC-93269) is a 2+1 BCMAxCD3 BsAb with bivalent binding to BCMA aimed at increasing its efficacy, a low CD3-binding domain, and a modified Fc region, mediating both reduced dosing and lower rates of cytokine release. The first-in-human phase 1 dose-finding study (NCT03486067) enrolled patients with TCE RRMM who had received at least three prior regimens. IV and SC alnuctamab were investigated; IV alnuctamab was administered at target doses of 0.15–10 mg with both fixed and step-up dosing; SC alnuctamab was administered with two step-up doses and target doses of 10, 15, 30, or 60 mg. Seventy patients received fixed or step-up doses of IV alnuctamab; the ORR was 39%, the mDoR was 33.6 months, and the mPFS was 3.1 months (36.6 months in responders). However, IV administration was associated with a high rate of CRS (76% of patients had a CRS event, including 7% with grade ≥ 3). Due to the high frequency of CRS, the trial pivoted to the SC administration of alnuctamab. Seventy-three patients received SC alnuctamab in dose escalation and dose expansion. The ORR was 54% across all doses, 63% at target doses ≥ 30 mg, and 69% (with 43% of at least CR) at the 30 mg target dose. The mPFS was 10.1 months across all dose levels and was not reached at a median follow-up of 9.3 months for the 30 mg target dose, with a 12-month PFS of 53%. Among the 28 patients who achieved a response and had an evaluable MRD, 28 (100%) were MRD-negative (flow cytometry, sensitivity 10–5). Safety was improved, with 56% of patients having CRS, all grade 1 or 2. Infections were reported in 62% of patients, with only 16% grades 3–4 and no grade 5 [46,47].

### 4.6. ABBV-383

ABBV-383 (TNB-383B) is a fully human BCMAxCD3 BsAb with a bivalent BCMA domain, silenced-FC backbone, and low CD3 affinity to enhance the efficacy and potentially minimize off-target toxicity and CRS, so as to reduce the need for step-up dosing. The safety and clinical activity of ABBV-383 were evaluated in a phase 1 dose escalation and expansion study (NCT03933735) for patients with TCE RRMM and who had received at least three prior lines of therapy. The preliminary results on 124 patients showed that IV ABBV-383 monotherapy (dose escalation [0.025–120 mg], *n* = 73; dose expansion [60 mg], *n* = 51) administered once every three weeks, without any step dosing was well tolerated and was associated with promising preliminary antitumor activity. The most common hematologic treatment-emergent AEs were neutropenia (37%) and anemia (29%), while the most common non-hematologic treatment-emergent AEs were CRS (57%, grade ≥ 3, 2%) and fatigue (30%). ABBV-383 doses of 40 mg once every three weeks and 60 mg once every three weeks have been selected for further dose exploration and optimal dose selection. The ORR was 57%, including 28% of at least CR, for all efficacy-evaluable patients and was 68%, including 36% of at least CR, in the ≥40 mg dose escalation plus dose expansion cohorts [48]. Updated results on 220 patients, 55 patients treated at 40 mg and 61 patients at 60 mg, showed an ORR of 64%, including 27% of at least CR, at the 40 mg dose level, and an ORR of 60%, including 35% of at least CR, at the 60 mg dose level; the mPFS was 13.7 and 11.2 months, respectively. CRS occurred in 71% (0% grade ≥ 3) and 70% (2% grade ≥ 3) of patients at the 40 mg and 60 mg doses, respectively, while grade 3–4 infections occurred in 26% and 34% of patients, respectively [49].

### 4.7. REGN-5459

REGN5459 is a BCMAxCD3 BsAb with a low affinity for CD3, an aspect that can be useful in attenuating CRS and reducing T-cell depletion. In the first-in-human phase 1/2 trial (NCT04083534), REGN-5459 was administered at doses of 3–60 mg or 120–900 mg weekly (with step-up dosing) to patients with TCE RRMM who had received at least three prior lines of therapy. Preliminary data on 43 patients with a median of five prior lines of therapy showed early and durable response with a manageable side-effect profile. The maximum tolerated dose was not reached, but the RP2D was 480 mg. Grade 3–4 hematologic AEs included neutropenia (37%), anemia (26%), and thrombocytopenia (19%). CRS occurred in 53.5% of the patients (most cases of grade 1, one case of grade 2, and two cases of grade 3), while one patient developed grade 2 ICANS. Infections occurred in 61% of patients (37% grade ≥ 3). The ORR was 65%, including 51% of at least CR, and the probability of maintaining a response at 12 months was 78%. Interestingly, the ORRs improved as the dose of REGN5459 increased: from 23% with 3–60 mg to 67% with 120–240 mg and 90.5% with 480–900 mg [50].

## 5. Non BCMA-Targeting BsAbs

Despite advances in therapy, including BCMA-targeting agents, patients still experience cycles of remission and relapse, and new therapies with different targets are needed.

Talquetamab is a first-in-class GPRC5DxCD3 BsAb and is currently the only non-BCMA-targeting BsAb approved for the treatment of adult patients with TCE RRMM after four (FDA) or three (EMA) prior therapies [11]. Further agents in advanced stages of clinical development include the GPRC5D-targeting BsAb forimtamig [51] and the FcRH5-targeting BsAb cevostamab [52] (Table 3).

### 5.1. Talquetamab

Talquetamab is a GPRC5DxCD3 IgG-like BsAb with a proline–alanine–alanine scaffold designed to minimize Fc-receptor binding. It was initially studied in the phase 1/2 MonumenTAL-1 trial (NCT03399799) that enrolled RRMM patients with progression or intolerance to all established therapies (phase 1), or those with TCE disease and who had received at least three prior lines of therapy (phase 2). Two RP2Ds, 0.4 mg/kg SC every week (with two step-up doses) and 0.8 mg/kg SC every other week (with three step-up doses), were identified [11]. Among the T-cell-redirection therapy-naïve population, 143 patients received talquetamab at the dose of 0.4 mg/kg, and 145 patients received the 0.8 mg/kg dose, while 70 patients were included in the prior T-cell-redirection therapy cohort and treated with either 0.4 mg/kg or 0.8 mg/kg dose. Among the T-cell-redirection therapy-naïve cohorts, the median number of prior lines of therapy was 5 (range, 2 to 17), and 74% and 69% of the patients were TCR in the 0.4 mg/kg and 0.8 mg/kg cohorts, respectively. At a median follow-up of 18.8 months, the 0.4 mg/kg dose level had an ORR of 74%, including 34% of at least CR, with mDoR and mPFS of 9.5 and 7.5 months, respectively. At a median follow-up of 12.7 months, the 0.8 mg/kg dose level achieved an ORR of 72%, including 39% of CR or better; the mDoR was not reached, while the mPFS was 14.2 months. The ORRs in high-risk subgroups were consistent with the overall population (being ≥60% for patients aged over ≥75 years, with high-risk cytogenetics, renal impairment, or ISS stage III) except in the EMD subgroup, although >40% of patients with EMD across the 0.4 mg/kg and 0.8 mg/kg cohorts demonstrated a response [53]. Among the prior T-cell-redirection therapy cohort, the median number of prior lines of therapy was six, and 84% of the patients were TCR. At a median follow-up of 14.8 months, the prior T-cell-redirection group had an ORR of 67% (41.5% of at least CR), being 73% among patients previously exposed to anti-BCMA CAR T-cell therapy and 56.5% among patients with prior anti-BCMA BsAb treatment. The twelve-month DoR and PFS were 54.7% and 50% in patients exposed to prior BCMA CAR-T, and 43.3% and 30.4% in patients exposed to prior BCMA BsAb [11,54,55]. Most high-grade AEs were cytopenias, with an incidence of grade 3–4 anemia of 31.5% in the 0.4 mg/kg cohort, 28% in the 0.8 mg/kg cohort, and 27.5% in the prior T-cell-redirection therapy cohort; grade 3–4 neutropenia at 31%, 22%, and 53%, respectively, in the three cohorts; and grade 3–4 thrombocytopenia at 20%, 19%, and 29%, respectively, in the three cohorts. The incidence of CRS was similar in the three cohorts, ranging from 74.5% to 79%, mainly grades 1–2 with less than 2% grade ≥ 3, while ICANS occurred in 11% of patients in the 0.4 mg/kg and 0.8 mg/kg cohorts, and 3% in the prior T-cell-redirection therapy cohort. All grades infections were observed in 59% of the patients (20% grades 3–4) in the 0.4 mg/kg cohort, 66% (14.5% grades 3–4) in the 0.8 mg/kg cohort, and 72.5% (27.5% grades 3–4) in the prior T-cell-redirection therapy cohort. Notably, the lower expression of GPRC5D on normal PCs than MM PCs may explain the differences in the frequency of infections between GPRC5D-targeting BsAbs and those targeting BCMA. However, anti-GPRC5D therapy is associated with on-target off-tumor AEs, deriving from the expression of GPRC5D on non-hematopoietic tissues. Indeed, skin-related events were reported in 56% of patients in the 0.4 mg/kg cohort, 73% in the 0.8 mg/kg cohort, and 69% in the prior T-cell-redirection therapy cohort (all but one grades 1–2), and nail-related events were found in 54.5% of patients in the 0.4 mg/kg cohort, 54% in the 0.8 mg/kg cohort, and 63% in the prior T-cell-redirection therapy cohort (all grades 1–2). Also, 72% of patients in the 0.4 mg/kg cohort, 71% in the 0.8 mg/kg cohort, and 76.5% in the prior T-cell-redirection therapy cohort had dysgeusia [11,54].

The impact of reducing the dose intensity on safety and efficacy is an area of clinical interest. The MonumenTAL-1 trial included two prospective dose intensity reduction cohorts. Twenty-four patients were included in the prospective cohorts; in total, 9 out of 12 patients achieved at least a PR and switched from 0.8 mg/kg every other week to 0.4 mg/kg every other week dosing, and 10 out of 12 patients achieved at least a PR and switched from 0.8 mg/kg every other week to 0.8 mg/kg monthly dosing. Prospective cohorts demonstrated maintenance of response following the switch, a trend for improved immune fitness, and a trend for improved resolution of GPRC5D-related oral, skin (rash and non-rash), and nail toxicities. Overall, these data support the flexibility to adjust the dosing of talquetamab in responders to potentially improve patient experience while maintaining efficacy [56].

### 5.2. Forimtamig

Forimtamig (RG6234) is a GPRC5D T-cell-engaging BsAb with a novel 2+1 (GPRC5D:CD3) configuration, aimed at increasing its efficacy, and a silent Fc region [51]. A phase 1 trial (NCT04557150) is evaluating the safety and clinical activity of IV or SC administration of forimtamig in patients with RRMM. Overall, 51 patients received 18–10,000 µg of IV forimtamig, and 57 patients received 1200−7200 µg of SC forimtamig preceded by step-up doses. The patients had medians of five and four prior lines of therapy in the IV and SC cohorts, respectively, 67% of the patients had TCR disease, 21% had prior anti-BCMA exposure, 47% had high-risk cytogenetics, and 30% had EMD. The preliminary data showed a high response rate across all tested doses. Specifically, in the IV cohort, the ORR was 71%, including 35% of patients with a CR or better, and the mDoR was 10.8 months, while in the SC cohort, the ORR was 64%, including 25.5% of patients with at least CR, and the mDoR was 12.5 months. Of the 21 patients with prior anti-BCMA exposure in the IV (*n* = 10) and SC (*n* = 11) cohorts, 5 (50%) and 6 (55%) had a response, respectively. AEs deriving from the use of forimtamig were consistent with the target class of anti-GPRC5D BsAbs. Infections were observed in 61% of patients (22% grade ≥ 3) in the IV cohort, and in 46% (26% grade ≥ 3) in the SC cohort. CRS was observed in 82% of patients in the IV cohort and 79% in SC cohort (only 2% grade ≥ 3 in both cohorts), while ICANS occurred in 10% of patients (2% grade ≥ 3) and 12% (4% grade ≥ 3), respectively. Hematologic AEs were common in both cohorts, with grade ≥ 3 anemia in 16% and 39% of the patients, neutropenia in 12% and 16%, and thrombocytopenia in 14% and 19% in the IV and SC cohorts, respectively. AEs related to GPRC5D expression on non-myeloma cells were mostly grades 1–2, including skin-related AEs (78% in IV cohort and 86% in SC cohort), mucosal AEs (73% in IV cohort and 77% in SC cohort), and hair or nail changes (23.5% in IV cohort and 28% in SC cohort).

### 5.3. Cevostamab

Cevostamab is an FcRH5xCD3 IgG-like BsAb. Initial data from the dose-escalation phase of the ongoing phase 1 study (NCT03275103) of cevostamab monotherapy in patients with heavily pretreated RRMM demonstrated promising activity and manageable safety [52]. Overall, 161 RRMM patients, for whom no established therapy was available or appropriate, received IV cevostamab in 21-day cycles for a total of 17 cycles, unless progressive disease or unacceptable toxicity occurred. The median prior lines of therapy was six, 85% of the patients had TCR, 17.5% had received prior CAR-T, 8% had prior BsAb, 17% had prior ADC, and 34% had a prior anti-BCMA targeting agent. At higher dose levels of 132–198 mg (*n* = 60 patients), the ORR was 57%, including CR in 8%. At target dose levels > 90 mg, the ORRs in patients with prior exposure to CAR-Ts, BsAbs, ADCs, and anti-BCMA targeting agents were 44% (4/9 patients), 33% (3/9), 50% (7/14), and 36% (8/22), respectively. The mDoR was 11.5 months. The most common AEs were CRS (80%, with only 1% being grade 3), cytopenias, and infections [52]. Preliminary data on 16 patients who achieved at least a PR, or better, by cycle 17 and maintained a response throughout that cycle showed that 13 of the 16 patients remained in remission, with 8 patients maintaining a response ≥6 months after completion of therapy, highlighting the potential for an extended treatment-free period following fixed-duration therapy [57]. Moreover, a dedicated tocilizumab pretreatment arm was added to determine whether a single dose of tocilizumab administered prior to the first dose of cevostamab can reduce the incidence of CRS. The incidence and severity of CRS in the 31 patients who received tocilizumab pretreatment were compared with the incidence and severity reported in 44 patients treated with cevostamab without tocilizumab. Prophylactic tocilizumab (8 mg/kg) efficiently reduced the overall incidence of CRS from 91% (40 of 44 patients) to 39% (12 of 31 patients), with no negative impact on disease response, although the incidence of grade 3–4 neutropenia was higher [58].

## 6. Using BsAbs in Combination Strategies and in Earlier Lines of Therapy

The use of BsAbs in combination strategies and/or application in earlier lines of therapy has the rationale to exploit a reduced tumor burden and better T-cell fitness, with the aim to further optimize treatment and increase its potency. At present, several trials are exploring different BsAbs combined with various anti-MM agents—including IMiDs, PIs, anti-CD38 mAbs, and/or anti-PD1 inhibitors—or with agents that enhance the expression of target antigens or are evaluating the combination of BsAbs directed against different targets. Preliminary data from early phase studies in RRMM are already available (Table 4).

The anti-CD38 mAb daratumumab may have synergism with BsAbs. CD38 is a cell-surface glycoprotein with a dual receptor and ectoenzyme activity. Its functions include receptor-mediated adhesion and signaling, enzymatic activities including intracellular calcium mobilization, and the production of adenosine, which is important for the induction of local immunological tolerance and implicated in the local survival strategy of neoplastic PCs in the bone marrow milieu. CD38 has a low level of expression on lymphoid and myeloid cells under normal conditions, and high level of expression on malignant cells in MM. Therefore, anti-CD38 mAbs may induce cellular death through various mechanisms, including CDC, ADCC, antibody-dependent cellular phagocytosis (ADCP), induction of apoptosis via cross-linking, and immunomodulation by means of depletion of CD38-positive regulatory T, regulatory B, and myeloid-derived suppressor cells (MDSCs), which in turn leads to an increase in T-helper cells, cytotoxic T cells, T-cell functional response, and T-cell receptor clonality [59]. Based on these characteristics, anti-CD38 mAbs have a key role in the treatment of MM and may also have a synergistic effect when combined with BsAbs, potentially counteracting the immune system dysfunction and improving the response. The TRIMM-2 study (NCT04108195) is a phase 1b multicohort study with the aim of evaluating teclistamab and talquetamab plus daratumumab, with or without pomalidomide. Preliminary results for the teclistamab-daratumumab combination, tested in 65 heavily pretreated RRMM patients with five median prior lines of therapy, 75% TCR, 12% with prior anti-BCMA exposure, and 63% anti-CD38 mAb refractory, showed a high ORR of 76.5%, including 21.5% of ≥CR, and a 74% ORR was also confirmed in patients with prior exposure to an anti-CD38 mAb. CRS was reported in 65% of the patients (all grades 1–2), neutropenia in 51% (46% grades 3–4), anemia in 46% (30% grades 3–4), thrombocytopenia in 32% (all grades 3–4), and pyrexia in 24% (all grades 1–2) [60]. Similarly, the combination of talquetamab–daratumumab was evaluated in 65 RRMM patients, of whom 14 patients were treated with talquetamab at 0.4 mg/kg every week and 51 patients with talquetamab at 0.8 mg/kg every other week. The median number of prior lines was five, 57% and 61% of the patients were TCR in the 0.4 mg/kg and 0.8 mg/kg dose cohorts, 57% and 53% had prior anti-BCMA exposure, and 79% and 78% were anti-CD38 mAb refractory, respectively. With the dose cohort of 0.8 mg/kg every other week, the ORR was 84%, including 52% of CR or better, the mPFS was 19 months, and the 12-month OS rate was 92%. CRS was reported in 80% of the patients (only grades 1–2), anemia in 49% (25.5% grades 3–4), neutropenia in 39% (27.5% grades 3–4), thrombocytopenia in 37% (20% grades 3–4), pyrexia in 37% (4% grades 3–4), skin AEs in 84% (8% grades 3–4), nail AEs in 69% (2% grades 3–4), and oral AEs in 90% (4% grades 3–4) [61]. Overall, these results suggest that combined immunomodulatory actions can yield robust responses in patients with refractory disease, including those who are refractory to anti-CD38/BCMA and T-cell-redirecting therapy. However, due to the low number of patients included in this trial, the published data on BsAbs plus anti-CD38 mAb combinations are still very preliminary; therefore, more data from ongoing trials are required before any recommendations can be made on when and how such regimens should be used.

Moreover, combining IMiD with T-cell-redirection therapy may lead to a synergistic anti-MM action due to a direct on-tumor apoptotic effect and enhanced immune activity. MonumenTAL-2 (NCT05050097) is a multi-arm, phase 1b study of talquetamab in combination with anti-MM agents in patients with RRMM treated with at least two prior lines of therapy including a PI and an IMiD. Preliminary results for the combination of talquetamab–pomalidomide showed promising efficacy and a manageable safety profile in 35 patients (16 patients in the 0.4 mg/kg weekly talquetamab dose cohort and 19 in the 0.8 mg/kg every other week cohort), with a median of three prior lines of therapy, 31% (0.4 mg/kg cohort) and 21% (0.8 mg/kg cohort) TCR, 25% (0.4 mg/kg) and 0% (0.8 mg/kg) with prior anti-BCMA T-cell-redirection therapy, and 31% (0.4 mg/kg) and 16% (0.8 mg/kg) pomalidomide exposed. The ORR was 94%, including 62.5% of at least CR, in the 0.4 mg/kg cohort, and 84%, including 37% of at least CR, in the 0.8 mg/kg cohort, with robust responses also observed in patients with prior exposure to CAR T-cell therapy or pomalidomide. CRS occurred in 74% of the patients (3% grades 3–4), neutropenia in 63% (54% grades 3–4), anemia in 37% (26% grades 3–4), thrombocytopenia in 29% (20% grades 3–4), infections in 80% (23% grades 3–4), skin AEs in 74% (6% grades 3–4), nail AEs in 69% (only grades 1–2), and taste related in 86% [62].

Based on the synergism of BsAbs with anti-CD38 mAbs and IMiDs, the three-drug combination of teclistamab–daratumumab–lenalidomide is under investigation in the phase 1b multicohort MajesTEC-2 trial (NCT04722146) for RRMM patients treated with 1–3 prior lines of therapy including an IMiD and a PI. Interestingly, this combination may result in an enhanced efficacy by exploiting the cytotoxic and immunomodulatory action of each molecule involved, while use in earlier lines of therapy could take advantage of a more favorable immune profile. Initial results in 32 patients with a median of two prior lines of therapy, 100% IMiD exposed, 28% lenalidomide refractory, and 19% anti-CD38 mAb refractory showed rapid responses that deepened over time. The ORR was 93.5%, including 55% of at least CR. Infections were reported to be the most common AEs, with a rate of 90.6% (grades 3–4 in 35.5% of patients). CRS occurred in 81% of the patients (all grades 1–2), neutropenia in 84% (78% grades 3–4), thrombocytopenia in 25% (16% grades 3–4), and anemia in 22% (12.5% grades 3–4) [63].

In addition, combinations of two BsAbs with different targets are under investigation, with the aim of overcoming some of the resistance mechanisms associated with monotherapy. The phase 1b RedirecTT-1 trial (NCT04586426) is evaluating the combination of teclistamb plus talquetamab in TCE RRMM patients with progression or intolerance to all established therapies. Preliminary data on 93 patients, 34 treated with the recommended phase 2 regimen (RP2R) of teclistamab at 3 mg/kg plus talquetamab at 0.8 mg/kg every other week, showed encouraging results. The median prior lines of therapy was four, 78% of the patients were TCR, 33% had high-risk cytogenetics, and 38% had EMD. With the RP2R, the ORR was 96%, including 41% of CR or better, and the 9-month PFS rate was 77%. Notably, a high ORR of 87.5% (29% of at least CR) and mPFS of 9.9 months were also reported in patients with EMD, suggesting that a dual-targeting approach that can intercept escape phenomena may have a role in aggressive diseases, usually characterized by higher clonal heterogeneity. The safety profile of the combination was consistent with that of monotherapies with no new or additive toxicity seen for either teclistamab or talquetamab. With the RP2R, CRS occurred in 73.5% of the patients (all grades 1–2), infection in 79% (38% grades 3–4), neutropenia in 56% (44% grades 3–4), anemia in 32% (24% grades 3–4), thrombocytopenia in 32% (24% grades 3–4), skin toxicity in 53%, nail toxicity in 41%, and dysgeusia in 47% [61]. With the same rationale, a phase 1 dose-escalating study of JNJ-5322, an IgG1 tri-specific antibody that binds to CD3 on T cells and BCMA and GPRC5D on MM cells, is ongoing in patients with RRMM (NCT05652335). Dual-antigen targeting may enhance the tumor response by circumventing tumor heterogeneity and antigen loss and improving the potency due to an increased antigen-binding avidity. In vitro, JNJ-5322 induced potent and dose-dependent cytotoxicity with concomitant T-cell activation only in MM cell lines that expressed one or both target proteins (BCMA, GPRC5D). In vivo, JNJ-5322 induced a potent antitumor activity in models that expressed one or both target proteins [64].

Moreover, the PD-1 signaling pathway plays a key role in immune checkpoint regulation, and the upregulation of immune checkpoint molecules is a potential mechanism of resistance developed by MM PCs to evade cytotoxic T-cell responses. Indeed, a baseline immune profile suggestive of T-cell dysfunction/exhaustion and immune suppression—including higher expression of PD-1—has been associated with a reduced response after treatment with BsAbs, providing the rationale for exploring combinations including checkpoint inhibitors, as is currently being evaluated in the TriMM-3 (NCT05338775) study [65]. Additionally, pharmacologic modulation to increase antigen expression is also an attractive strategy, and the utilization of γ-secretase inhibitor at low doses to increase BCMA expression is under investigation (NCT04722146, NCT05090566, and NCT05259839). Finally, due to the favorable results of BsAbs in heavily pretreated RMM and the relevance of T-cell fitness in the effectiveness of T-cell-redirecting therapy, ongoing phase 3 clinical trials are currently evaluating BsAb monotherapy, or in combination, compared with standard therapies, as a treatment of RRMM in earlier lines of treatment, or as a first-line treatment of newly diagnosed MM (NDMM) patients (Table 5).

More in detail, the MajeTEC-3 trial (NCT05083169) is comparing teclistamab–daratumumab versus daratumumab–pomalidomide–dexamethasone (DaraPd) or daratumumab–bortezomib–dexamethasone (DaraVd) in RRMM patients treated with 1–3 prior lines of therapy (and refractory to lenalidomide if 1 prior line), while teclistamab monotherapy versus pomalidomide–bortezomib–dexamethasone (PVd) or carfilzomib–dexamethasone (Kd) is under investigation in the MajesTEC-9 trial (NCT05572515) enrolling RRMM patients treated with 1–3 prior lines of therapy including lenalidomide and an anti-CD38 mAb. Similarly, the MaonumenTAL-3 trial (NCT05455320) is evaluating talquetamab–daratumumab–pomalidomide versus talquetamab–daratumumab versus DaraPd in RRMM patients treated with at least 1 prior therapy including lenalidomide and a PI, and in a similar setting, the MagnetisMM-5 trial (NCT05020236) is evaluating elranatamab versus elranatamab–daratumumab versus DaraPd, while the LINKER-MM3 trial (NCT05730036) is comparing linvoseltamab versus elotuzumab–pomalidomide–dexamethasone (EloPd) upon 1 to 4 prior lines of therapy including lenalidomide and a PI. Among NDMM patients not intended for autologous stem cell transplantation (ASCT), the combinations of teclistamab–lenalidomide–dexamethasone versus talquetamab–lenalidomide–dexamethasone versus daratumumab–lenalidomide–dexamethasone (DaraRd) are under investigation in the context of the MajesTEC-7 trial (NCT05552222), while the MagnetisMM-6 trial (NCT05623020) is comparing elranatamab–lenalidomide–dexamethasone versus DaraRd. Lastly, BsAbs are under investigation as new post-ASCT maintenance therapies in the MajesTEC-4/EMN30 trial (NCT05243797) with teclistamab versus teclistamab–lenalidomide versus lenalidomide and in the MagnetisMM-7 trial (NCT05317416) with elranatamab versus lenalidomide.

## 7. Toxicity and Management

The main toxicities of BsAbs, in terms of incidence and/or clinical impact, include CRS, ICANS, cytopenias, and infections. Moreover, skin and nail disorders and dysgeusia can occur with GPRC5D-targeting BsAbs.

CRS is a systemic inflammatory reaction resulting as a consequence of robust cytotoxic T-cell activation and the subsequent release of inflammatory cytokines, particularly IL-6, IL-2, IFN-γ, and TNF. The clinical presentation varies from isolated fever to severe reactions including hypotension and hypoxemia. CRS has been reported with all BsAbs currently in clinical development, with an incidence ranging approximately between 40% and 80%; however, most events are low grade (with less than 2% being grade ≥ 3 CRS), and generally confined to the step-up doses or first full dose [7,8,11,32,33,44,45,46,47,48,49,50,51,52]. The variability in the number of step-up doses, use and schedule of pre-medications, route of administration, and differences in CD3-binding affinities among different BsAbs can partly explain some differences in CRS frequency. Anyway, patients should be carefully monitored during BsAb therapy for signs and symptoms of CRS to allow for early intervention, and inpatient hospital monitoring during step-up or the first full dose, or both, was required in the initial clinical trials. Recommendations for the management of CRS, when it occurs, include supportive care and the early administration of tocilizumab; if CRS persists or recurs after 1–3 doses of tocilizumab, second-line therapy (i.e., steroids) can be started. Prophylactic use of tocilizumab prior to BsABs is being evaluated, showing encouraging results [39,40,58]; such results are noteworthy, as these findings could likely facilitate the initiation of outpatient therapy in patients with suitable profiles, with significant relief on the burden of patients requiring hospitalization. However, the use of tocilizumab as prophylaxis is currently considered investigational and not recommended in clinical practice [66,67,68].

ICANS can be triggered by the passive diffusion of cytokines and trafficking of T cells into the central nervous system, monocyte recruitment, and macrophage activation. It is often preceded by CRS, which appears to be a triggering event or cofactor and typically consists of varying degrees of diffuse encephalopathy with or without focal signs, intracranial hypertension, or seizures [66]. Its incidence after BsAbs therapy is mostly less than 10–15%, with rare high-grade cases [7,8,11,32,33,44,45,46,47,48,49,50,51,52]. The management of ICANS primarily relies on the use of corticosteroids, with dexamethasone being the preferred initial treatment [66,67,68].

Cytopenias are commonly reported across all clinical trials of BsAbs, including neutropenia (grade ≥ 3 20–65%), anemia (grade ≥ 3 20–40%), thrombocytopenia (grade ≥ 3 10–25%), and lymphopenia (grade ≥ 3 15–35%) [7,8,11,32,33,44,45,46,47,48,49,50,51,52]. The underlying etiology is poorly understood, but the cytokine storm or milieu associated with CRS probably contributes to bone marrow suppression. Cytopenias appear more common earlier in treatment and seem to be more common with the BCMA-targeting BsAbs than with the non-BCMA-targeting agents. These hematological toxicities can be managed with dose delays and supportive care strategies. Growth factor use has been generally allowed in patients with grade ≥ 3 neutropenia, although not during active CRS [66,67,68].

Infections are common complications seen in patients treated with BsAbs and can be partially explained by ongoing T-cell activation, T-cell exhaustion, hypogammaglobinemia, and neutropenia. According to a pooled analysis of more than 1000 patients treated with BsAbs, infections affected more than half of the patients, including a quarter of grade 3–4 events, mainly represented by upper and lower respiratory tract infections, as well as opportunistic infections such as CMV, adenovirus, PJP, and aspergillosis [69]. Differences in the incidence and severity reported by clinical trials could account for differences in the target, schedule, and duration of therapy as well as differences in the median follow-up [7,8,11,32,33,44,45,46,47,48,49,50,51,52]. BCMA-targeting BsAbs have been associated with a higher incidence of infection, with more severe grade 3–4 infections than non-BCMA BsAbs. Notably, recent data on BCMA BsAbs reported a reduction in the incidence of severe infections when switching from a once-weekly to a once-every-2-weeks dosing schedule, suggesting that a less intensive schedule might mitigate the infection risk [8,32]. Preventive measures against infection include vaccination and drug prophylaxis, particularly with aciclovir or valaciclovir and co-trimoxazole, while antifungal and antibiotic prophylaxis are recommended only for patients with a high risk of infection. Moreover, prophylactic administration of IVIG is recommended in patients with low IgG concentrations (<400 mg/dL) or in patients with higher IgG concentrations and recurrent bacterial infections who do not respond to antibiotic therapy [66,67].

On-target, off-tumor toxicities of GPRC5D-targeting BsAbs are related to the target’s expression on cells that are able to produce hard keratin structures, such as hair follicles, and salivary glands, and include dermatologic and oral AEs. Dysgeusia has been reported with an incidence ranging approximately between 45% and 75%, while skin and/or nail toxicity was reported to be between 25% and 75% [11,51]. Proper management, education, and supportive care help ensure that patients can stay on treatment and receive optimal benefits from talquetamab. Moreover, recent data from the MonumenTAL-1 trial, including the prospective dose intensity-reduction cohorts, suggest that switching to a less intensive schedule of talquetamab can be associated with a trend for the improved resolution of GPRC5D-related oral, skin (rash and non-rash), and nail toxicities [56].

## 8. Discussion and Conclusions

Major improvements in the understanding of disease biology and tumor microenvironment have led to the introduction of novel, highly effective immunotherapies in the treatment landscape of MM. Two BCMA-targeting (teclistamab and elranatamab) plus one GPRC5D-targeting (talquetamab) BsAbs have achieved regulatory approval based on the impressive clinical activity showed in phase 2 non-randomized trials, and they currently represent new standards of care after the third or fourth line of therapy. Moreover, several newer agents, as well as combination strategies and use in different settings, are under active investigation.

Several clinical trials exploring the use of BsAbs as monotherapy in the context of heavily pretreated RRMM patients (median of 4–6 prior lines) have already shown deep responses. Indeed, the available data from these studies reported high rates of CR (ranging from 8% to 45.5% of patients) and high rates of MRD negativity (ranging from 54.3% to 100% of evaluable patients). Notably, these results ultimately translated into favorable survival outcomes, with mPFS of around 1 year (mPFS ranging between 7.5 and 17.2 months, when available) [7,8,11,32,33,44,45,46,47,48,49,50,51,52]. Similar efficacy data have been reported with different constructs and targets, with some differences in terms of the route of administration, frequency, need for step-up doses, and, therefore, logistic issues. Regarding toxicity, high-grade CRS and ICANS are observed only in a minority of patients treated with BsAbs, while cytopenias and severe infections, due to prolonged hypogammaglobulinemia and lymphodepletion, are frequently observed [7,8,11,32,33,44,45,46,47,48,49,50,51,52]. The risk of infection seems to be higher among patients treated with BCMA-targeting BsAbs than non-BMCA-targeting BsAbs, likely due to the different expression of the targets on B-lymphocytes and normal PCs and the subsequent different B-cell depletion levels and grades of hypogammaglobulinemia. On the other hand, anti-GPRC5D-targeting BsAbs correlate with on-target off-tumor toxicities, including skin and nail disorders and dysgeusia, due to the expression of this antigen in the skin and keratinized tissue. Notably, the efficacy and safety outcomes reported by clinical studies have been further confirmed by real-world data, including a broader population of patients, characterized by different backgrounds in terms of the number and types of previous therapies, and with more aggressive disease and comorbidities [36,37,38].

Strategies to optimize the use of BsAbs treatments may aim to improve their tolerance and reduce their toxicity, as well as enhance their potency. Recommended measures to prevent and manage AEs include premedication and step-up dosing, tocilizumab and corticosteroids in the case of CRS, as well as baseline and periodic testing for specific infections and the incorporation of prophylactic measures, including supportive therapy with IVIG. Moreover, modifications in treatment schedules with reduced dose intensities for responder patients, as well as fixed durations of therapy, may probably reduce the risk of AEs and improve the quality of life and patient adherence, all while maintaining the efficacy [34,56,57]. Recently, preclinical data have shown that treatment-free intervals may also be beneficial to preserve T-cell fitness and improve the antitumor activity; moreover, even if treatment-free intervals do not affect the efficacy, they are likely to reduce the frequency and severity of infections [70]. Meanwhile, strategies to further increase the anti-MM activity include the use of BsAbs in combination regimens as well as in earlier lines of therapy. Early phase studies have reported favorable results with the use of BsAbs combined with anti-CD38 mAbs and/or IMiDs due to the direct on-tumor apoptotic effect and enhanced immune activity [60,61,62,63]. In addition, combining BsAbs targeting different PCs antigens or the use of tri-specific antibodies may improve potency further while evading tumor heterogeneity and antigen loss. At the same time, a more tumor-targeted delivery by binding to two MM-associated antigens may also lead to lower ‘on-target/off-tumor’ toxicity [61,64]. Moreover, BsAbs are increasingly being used in earlier lines of therapy as induction, consolidation, or maintenance, aiming to potentially leverage better T-cell health, a lower tumor burden, less clonal evolution, and better functional status. Indeed, although the mechanisms of resistance to BsAbs have yet to be unraveled and few data are available, it has been reported that the biallelic loss of target or point mutations, T-cell exhaustion, lack of recruitment or priming of T cells, and the immunosuppressive bone marrow microenvironment play a potential role [71,72].

In addition to BsAbs, others novel immunotherapy approaches include ADCs and CAR T-cells. While the BCMA-targeting ADC (belantamb mafodotin) is now no longer available following the market withdrawal [73,74], two BCMA-directed CAR T-cell products (ide-cel and cilta-cel) are available for the management of heavily pretreated patients with RRMM [9,10]. Therefore, patient-, disease-, and treatment-related factors should be considered to select the most suitable therapeutic approach. Belantamab mafodotin has shown a lower efficacy when compared with T-cell-redirecting therapies, with approximately one-third of responsive patients and a median PFS of about 3 months; on the other hand, it has a good safety profile, with the main toxicity represented by ocular AEs (reported in 71% of patients, including grade ≥ 3 keratopathy in 27%), and has the logistic advantage of a 3-week interval administration not requiring hospitalization, resulting in a potentially suitable treatment for frail patients, provided there is close monitoring for ophthalmological side effects [73,74]. Differently, BsAbs and CAR T-cell therapy have shown high rates of deep and durable responses, but these T-cell-redirecting therapies may be limited by life-threatening AEs, thereby requiring a multidisciplinary teamwork approach. Albeit cross-trial comparison is limited by multiple factors, the ORR, CR rates, and PFS of BsAbs used as monotherapy seem to be lower and shorter when compared with CAR T-cell products (ORR of 98%, including 83% of patients who achieved at least CR, and mPFS of 34.9 months reported for cilta-cel in the CARTITUDE-1 trial) [10,75]; however, promising results have been reported in the case of combination strategies, leading to results similar to those reported with CAR T-cell-based therapies. On the other hand, BsAbs seem to be associated with a lower grade of acute toxicities than CAR-T. Indeed, high-grade CRS and ICANS can occur after CAR T-cell therapy in up to 5% of patients, but this risk is lower with BsAbs and mainly limited to the first few doses, while severe infections can occur in up to 20% of patients after CAR T-cell therapy and in more than 50% of patients after BsAbs due to the prolonged hypogammaglobulinemia and lymphodepletion. Lastly, CAR T-cell therapies are hampered by the complexity of multi-step processes, ultimately affecting the percentage of patients who have actual access to the final product mainly due to disease progression and loss of eligibility criteria [9,10]; by contrast, off-the-shelf treatments based on ADCs and BsAbs can be quickly provided to patients. Collectively, these novel immunotherapies harbor important differences in terms of efficacy, safety, accessibility, and overall management. Identifying the best candidates for either treatment is therefore fundamental to maximizing its efficacy and improving patient prognosis.

Meanwhile, how to combine, or sequence, these therapies, are issues to be addressed. Preliminary data deriving from sub- or pooled analyses, specific cohorts, and real life suggest that retreatment with another type of novel immunotherapy can be currently considered as an effective therapeutic strategy. BCMA retreatment with different agents can maintain the effectiveness, albeit with a reduced ORR, and the wash-out time might potentially be an issue, especially for BsAbs before other BsAbs or CAR-T therapies, due to the risk of T-cell exhaustion [33,41,76,77]. Moreover, the use of anti-GPRC5D agents after anti-BCMA treatments can be feasible and effective, even if a reduced ORR seems to be achieved when two BsAbs with different targets are used in succession [11,51,52,54,55]. The availability of non-BCMA-targeting BsAbs may provide options for patients who have experienced a suboptimal response and/or antigen loss with BCMA-targeting agents, or for patients who may need treatment with a novel mechanism of action, preserving BCMA-targeting therapy for subsequent lines. Overall, the switch of the target and/or mechanism of action seems to be associated with better efficacy, while the use of BsAbs prior to CAR T-cell treatment seems to reduce the therapeutic efficacy; however, the therapeutic choice remains largely ‘empirical’ and not yet driven by mechanisms of resistance and levels of immune fitness/exhaustion.

Overall, the entry of BsAbs into the anti-myeloma therapeutic armamentarium has shown these molecules to be excellent candidates for inclusion in the anti-MM therapeutic backbone. Active research to increase their efficacy, improve their tolerance, and reduce toxicity is still in progress. A better understanding of the mechanisms of resistance is a challenge and, in the future, could guide clinicians to choose the best treatment and the most appropriate target, leading to an individualized approach.

## Figures and Tables

**Table 1:** Major targets in MM and corresponding BsAbs.

Target	Expression	Associated Signaling Pathway	Pathogenesis in MM	BsAbs
BCMA	Highly expressed on the surface of MM PCs and expressed at a low dose on B-lymphocytes and normal PCs; BCMA overexpression increases with disease progression	NF-κB, ERK1/2, MAPK8/JNK, AKT/PI3K, and STAT3 pathways	Survival Apoptosis Proliferation Angiogenesis Metastasis Migration	Pacanalotamab (AMG 420); teclistamab; elranatamab; linvoseltamab (REG 5458); REGN 5459; alnuctamab (CC-93269); ABBV-383; pavuratamab (AMG 701); TNB-383B; RO729089; WVT078; F182112
GPRC5D	Highly expressed on the surface of MM PCs; detected in immune cells predominantly with a PC phenotype and in cells producing hard keratin, such as cortical cells of the hair shaft, the keratogenous zone of the nail, and filiform papillae of the tongue	Unknown ligand and not-yet-established signaling mechanism and function		Talquetamab; forimtamig (RG6234, RO7425781)
FcRH5	Exclusively expressed in the B cell lineage, with increasing expression in mature B cells and PCs and higher expression on MM PCs than on normal PCs	High-affinity ligands and biological significance of FcRH5 are largely unknown		Cevostamab (BFCR4350A)

Abbreviations: BCMA = B-cell maturation antigen; MM = multiple myeloma; PC = plasma cells; NF-κB = nuclear factor kappa B; ERK1/2 = extracellular signal-related kinase; MAPK8/JNK = mitogen-activated protein kinase 8/c-Jun N-terminal kinases; AKT/PI3K = protein kinase B/phosphatidylinositol 3-kinase; STAT3 = signal transducer and activator of transcription 3; GPRC5D = G protein-coupled receptor class C group 5 member D; FcRH5 = Fc receptor-like 5.

**Table 2:** BCMA-targeting BsAbs.

BsAb	Teclistamab	Elranatamab	Linvoseltamab	Alnuctamab	ABBV-383	REGN-5459
Structure	Humanized Ab	Humanized Ab	Veloci-Bi^®^ platform, fully human Ab	Humanized Ab, bivalent binding	Low CD3 affinity, fully human Ab	Low CD3 affinity, fully human Ab
Clinical trials	Phase 1/2 trial MajesTEC-1 (NCT03145181; NCT04557098)	Phase 2 trial MagnetisMM-3 (NCT04649359, cohort A)	Phase 1/2 trial LINKER-MM1 (NCT03761108)	Phase 1 trial (NCT03486067)	Phase 1 trial (NCT03933735)	Phase 1/2 trial (NCT04083534)
Dosing schedule	0.06–0.3–1.5 mg/kg SC QW, switch to Q2W if ≥CR for 6 mo	12–32–76 mg SC QW, switch to Q2W from C7 if ≥PR	5–25–200 mg IV QW C1–C3, Q2W C4–C5, Q4W later if ≥VGPR	Target dose: 30 mg SC QW C1–C3, Q2W C4–C6, Q4W from C7	60 mg IV Q3W	Target dose: 480 mg IV QW
Pts n°	165	123	117 at 200 mg	73 30 at RP2D	220 55 at 40 mg 61 at 60 mg	43
Prior LOT, median n°	5	5	5	4	5	5
TCR, %	78	97	81	63	80	61
ORR, %	63	61	71	69 @30 mg	64 @ 40 mg 60 @60 mg	65
≥CR, %	45.5	35	30	43 @30 mg	27@ 40 mg 35 @60 mg	51
PFS	11.3 mo	17.2 mo	66% at 12 mo	53% at 12 mo @30 mg	13.7 mo @ 40 mg 11.2 mo @ 60 mg	NA
DoR	21.6 mo	71.5% at 15 mo	87% at 12 mo	64% at 12 mo	70% at 12 mo @ 40 mg 66% at 12 mo @ 60 mg	78% at 12 mo
OS	21.9 mo	21.9 mo	NA	NA	NA	NA
CRS (Gr 3–4), %	72 (0.6)	58 (0)	45 (1)	56 (0)	71 (0) @ 40 mg 70 (2) @ 60 mg	53.5 (5)
ICANS (Gr 3–4), %	3 (0)	3 (0)	6 (3)	3 (0)	5 (0) @ 40 mg 5 (2) @ 60 mg	2 (0)
Cytopenias (Gr 3–4), %					42 (31) @ 40 mg	
Neutropenia	71 (65.5)	49 (49)	32.5 (31)	45 (42)	43 (34) @ 60 mg	39.5 (37)
					55 (31) @ 40 mg	
Anemia	54.5 (38)	49 (37)	27 (24)	27 (25)	38 (13) @ 60 mg	35 (26)
					36 (16) @ 40 mg	
Thrombocytopenia	41 (22)	31 (24)	17 (14)	16 (14)	26 (13) @ 60 mg	23 (19)
Infections (Gr ≥ 3), %	80 (68)	70 (46)	60 (37)	62 (16)	71 (26) @ 40 mg 57 (34) @ 60 mg	61 (37)

Abbreviations: BCMA = B-cell maturation antigen; BsAbs = bispecific antibodies; Ab = antibody; SC = subcutaneous; QW = every week; Q2W = every other week; CR = complete response; mo = months; C = cycle; PR = partial response; IV = intravenous; Q4W = every 4 weeks; VGPR = very good partial response; Q3W = every 3 weeks; pts = patients; n° = number; RP2D = recommended phase 2 dose; LOT = line(s) of therapy; TCR = triple-class refractory; ORR = overall response rate; PFS = progression-free survival; DoR = duration of response; OS = overall survival; NA = not available; CRS = cytokine release syndrome; Gr = grade; ICANS = immune effector cell-associated neurotoxicity.

**Table 3:** Non-BCMA-targeting BsAbs.

BsAb	Anti-GPRC5D Talquetamab			Anti-GPRC5D Forimtamig		Anti-FCRH5 Cevostamab
Structure	Humanized Ab			Bivalent binding		Humanized Ab
Clinical trials	Phase 1/2 MonumenTAL-1 trial (NCT03399799)			Phase 1 trial (NCT04557150)		Phase 1 trial (NCT03275103)
Pts n°	143	145	70	51	57	161
Prior T-cell-redirecting tp exposure	Naïve (ADC allowed)	Naïve (ADC allowed)	Anti-BCMA (CAR-T/BsAb)	Anti-BCMA	Anti-BCMA	
Dosing schedule	0.4 mg/kg SC QW	0.8 mg/kg SC Q2W	0.4 mg/kg SC QW or 0.8 mg/kg SC Q2W	18–10,000 µg/kg IV Q2W/Q3W	1200–7200 µg/kg SC Q2W/Q3W	20–198 mg IV Q3W
Prior LOT, median n°	5	5	6	5	4	6
TCR, %	74	79	84	62	72	85
ORR, % ORR prior BCMA, %	74	72	67 73 prior CAR-T 56.5 prior BsAb	71 50	64	57
≥CR, %	34	39	41.5	35	25.5	8
PFS	7.5 mo	14.2 mo	44% at 12 mo	NA	NA	NA
DoR	9.5 mo	90.5% at 12 mo (pts with ≥CR)	55% at 12 mo	10.8 mo	12.5 mo	11.5 mo
OS	76% at 12 mo	78% at 12 mo	80% at 12 mo	NA	NA	NA
CRS (Gr 3–4), %	79 (2)	74.5 (0.7)	76.5 (2)	82 (2)	79 (2)	80 (1)
ICANS (Gr 3–4), %	11 (1.4)	11 (1.4)	3 (NA)	10 (2)	12 (4)	14 (0.6)
Cytopenias (Gr 3–4), %						
Neutropenia	35 (31)	28 (22)	55 (53)	24 (12)	18 (16)	39 (36)
Anemia	45 (31.5)	45.5 (28)	49 (27.5)	33 (16)	49 (39)	33 (22)
Thrombocytopenia	27 (20)	30 (19)	37 (29)	31 (14)	26 (19)	26 (18)
Infections (Gr ≥ 3), %	59 (20)	66 (14.5)	72.5 (27.5)	61 (22)	46 (26)	42.5 (19)
Dysgeusia, %	72	71	76.5	45	46	NA
Skin/nail toxicity, %	56/54.5	73/54	69/63	78/23.5	86/28	NA

Abbreviations: BCMA = B-cell maturation antigen; BsAbs = bispecific antibodies; GPRC5D = G protein-coupled receptor class C group 5 member D; FcRH5 = Fc receptor-like 5; Ab = antibody; pts = patients; n° = number; tp = therapy; ADC = antibody–drug conjugate; CAR-T = chimeric antigen receptor T-cell therapy; SC = subcutaneous; QW = every week; Q2W = every other week; Q3W = every 3 weeks; IV = intravenous; LOT = line(s) of therapy; TCR = triple-class refractory; ORR = overall response rate; CR = complete response; PFS = progression-free survival; mo = months; NA = not available; DoR = duration of response; OS = overall survival; CRS = cytokine release syndrome; ICANS = immune effector cell-associated neurotoxicity; Gr = grade.

**Table 4:** BsAb combinations.

BsAb Combination	Tec-Dara	Tal-Dara		Tal-Poma		Tec-Dara-Len	Tal-Tec
Clinical trial	Phase 1 trial TRIMM-2 (NCT04108195)			Phase 1b trial MonumenTAL-2 (NCT05050097)		Phase 1b trial MajesTEC-2 (NCT04722146)	Phase 1b trial RedirecTT-1 (NCT04586426)
Dosing schedule	Tec SC 1.5 mg/kg QW, 3.0 mg/kg Q2W, 3.0 mg/kg QW	Tal SC 0.4 mg/kg QW	Tal SC 0.8 mg/kg Q2W	Tal SC 0.4 mg/kg QW	Tal SC 0.8 mg/kg Q2W	Tal SC 0.72 or 1.5 mg/kg QW, 3.0 mg/kg from C3	Tec 3.0 mg/kg Tal 0.8 mg/kg SC Q2W
Pts n°	65	14	51	16	19	35	34
Prior LOT, median n	5	6	5	3	3	2	4
TCR, %	75	57	61	31	21	NA	76.5
Prior BCMA-targeted tp, %	12	57	53	25	0	NA	NA
Prior PI/IMiD/anti-CD38 mAb, %	NA/97/63	NA/NA/79	NA/NA/90	NA/NA/75	NA/NA/74	100/100/28.5	NA/NA/NA
ORR, %	76.5	71	84	94	84	93.5	96
≥CR, %	21.5	43	52	62.5	37	55	41
DoR	NA	NA	20 mo	100% at 9 mo	84% at 9 mo	NA	NA
PFS	NA	77% at 12 mo	19 mo	94% at 9 mo	75.5% at 9 mo	NA	77% at 9 mo
OS	NA	92% at 12 mo	91.5% at 12 mo	NA	NA	NA	NA

Abbreviations: BsAbs = bispecific antibodies; Tec = teclistamab; Dara = daratumumab; Tal = talquetamab; Poma = pomalidomide; Len = lenalidomide; SC = subcutaneous; QW = every week; Q2W = every-other week; C = cycle; pts = patients; n° = number; LOT = line(s) of therapy; TCR = triple-class refractory; BCMA = B-cell maturation antigen; tp = therapy; PI = proteasome inhibitor; IMiD = immunomodulatory drug; mAb = monoclonal antibody; NA = not available; ORR = overall response rate; CR = complete response; DoR = duration of response; mo = months; PFS = progression-free survival; OS = overall survival.

**Table 5:** Ongoing phase 3 trials evaluating the use of BsAb monotherapy, or in combination, compared with standard therapies.

Trial	Regimen	Study Population	Primary Endpoint	Recruitment Status	Estimated Date of Completion
Trials with Teclistamab					
MajesTEC-3 (NCT05083169)	Teclistamab + daratumumab vs. DaraPd or DaraVd	RRMM, 1–3 prior LOTs	PFS (up to 5 yrs)	Active, not recruiting	Dec 2028
MajesTEC-9 (NCT05572515)	Teclistamab vs. PVd/Kd	RRMM, 1–3 prior LOTs including R and anti-CD38	PFS (up to 9 yrs)	Recruiting	Aug 2031
MajesTEC-4/EMN30 (NCT05243797)	Teclistamab vs. teclistamab + lenalidomide vs. lenalidomide	ND after ASCT	PFS (up to 8 yrs)	Recruiting	Apr 2032
MajesTEC-7 (NCT05552222)	Teclistamab + daratumumab + lenalidomide vs. talquetamab + daratumumab + lenalidomide vs. DaraRd	NDMM, TNE	PFS (up to 9 yrs)	Recruiting	Oct 2033
Trials with Talquetamab					
MonumenTAL-3 (NCT05455320)	Talquetamab + daratumumab + pomalidomide vs. talquetamab + daratumumab vs. DaraPd	RRMM, ≥1 prior LOT including R and PI	PFS (up to 6 yrs 6 mos)	Recruiting	Sep 2029
Trials with Elranatamab					
MagnetisMM-5 (NCT05020236)	Elranatamab vs. elranatamab + daratumumab vs. DaraPd	RRMM, ≥1 prior LOT including R and PI	Part 1: DLT in the first 42 days after 1st dose Part 2: PFS (up to 51 mos)	Recruiting	Sep 2026
MonumenTAL-5 (NCT05461209)	Talquetamab vs. belantamab mafodotin	RRMM, ≥4 prior LOTs including anti-CD38 and double refractory	ORR (up to 1 yr 3 mos)	Not recruiting	Withdrawn for business decision
MagnetisMM-6 (NCT05623020)	Elranatamab + daratumumab + lenalidomide vs. DaraRd	NDMM, TNE	Part 1 DLT in the first 28/76 days after 1st dose Part 2: PFS (up to 73 mos) and sMRD-neg	Recruiting	Nov 2031
MagnetisMM-7 (NCT05317416)	Elranatamab vs. lenalidomide	NDMM after ASCT	PFS (up to 5 yrs)	Recruiting	Oct 2029
Trials with Linvoseltamab					
LINKER-MM3 (NCT05730036)	Linvoseltamab vs. EloPd	RRMM, 1–4 prior LOTs including R and PI	PFS (up to 5 yrs)	Recruiting	Dec 2026

Abbreviations: BsAbs = bispecific antibodies; NDMM = newly diagnosed multiple myeloma; RRMM = relapsed/refractory (R/R) multiple myeloma; LOTs = lines of therapy; PFS = progression-free survival; yrs = years; mos = months; R = lenalidomide; DaraPd = daratumumab + pomalidomide + dexamethasone; DaraVd = daratumumab + bortezomib + dexamethasone; PVd = pomalidomide + bortezomib + dexamethasone; Kd = carfilzomib + dexamethasone; TNE = transplant non-eligible; DaraRd = daratumumab + lenalidomide + dexamethasone; PI = protease inhibitor; DLT = dose-limiting toxicities; ORR = overall response rate; sMRD-neg = sustained minimal residual disease negativity; EloPd = elotuzumab + pomalidomide + dexamethasone.
